# Extensively Antibiotic-Resistant Bacterial Infections in Trauma Cases Managed at the Médecins Sans Frontières Tertiary Orthopaedic Center in Mosul, Iraq: A Case Series

**DOI:** 10.1093/ofid/ofae379

**Published:** 2024-07-08

**Authors:** Hisham Abdulrahman Ahmed, Humam Hasheem Mahmood, Haitham Hosam Aldin Sami, Abdullah Natiq Taher, Pilar Garcia-Vello, Engy Ali, Ernestina Repetto, Anita Williams, Fabiola Gordillo Gomez, Krystel Moussally

**Affiliations:** Tertiary Orthopaedic Center, Operational Centre Brussels, Médecins Sans Frontières, East Mosul, Iraq; Ministry of Public Health, Ninawa Governorate, Iraq; Macquarie University Hospital, Australian Orthopaedic Association, Sydney, Australia; Tertiary Orthopaedic Center, Operational Centre Brussels, Médecins Sans Frontières, East Mosul, Iraq; Ministry of Public Health, Ninawa Governorate, Iraq; Tertiary Orthopaedic Center, Operational Centre Brussels, Médecins Sans Frontières, East Mosul, Iraq; Tertiary Orthopaedic Center, Operational Centre Brussels, Médecins Sans Frontières, East Mosul, Iraq; Ministry of Public Health, Ninawa Governorate, Iraq; MSF Lebanon Branch Office, Middle East Medical Unit, Operational Centre Brussels, Médecins Sans Frontières, Beirut, Lebanon; Luxembourg Operational Research Unit, Medical Department, Operational Centre Brussels, Médecins Sans Frontières, Luxembourg; Directorate of Health, Ministry of Health Luxembourg, Luxembourg; Infectious Diseases Service, Saint Pierre University Hospital, Brussels, Belgium; MSF Lebanon Branch Office, Middle East Medical Unit, Operational Centre Brussels, Médecins Sans Frontières, Beirut, Lebanon; Luxembourg Operational Research Unit, Medical Department, Operational Centre Brussels, Médecins Sans Frontières, Luxembourg; MSF Lebanon Branch Office, Middle East Medical Unit, Operational Centre Brussels, Médecins Sans Frontières, Beirut, Lebanon; Medical Department, Operational Centre Brussels, Médecins Sans Frontières, Brussels, Belgium; MSF Lebanon Branch Office, Middle East Medical Unit, Operational Centre Brussels, Médecins Sans Frontières, Beirut, Lebanon

**Keywords:** access to antibiotics, extensive antibiotic resistance, Iraq, surgical management, trauma

## Abstract

The Médecins Sans Frontières Tertiary Orthopaedic Care center in Mosul, Iraq, provides reconstructive surgery, microbiological analysis, integrated infection prevention and control, and antibiotic stewardship services. Between May 2018 and February 2020, we recorded soft tissue and/or bone infections caused by gram-negative extensively drug-resistant (XDR) bacteria in 4.9% (13/266) of the admitted patients. The XDR bacteria identified among 12 patients in this case series were extended-spectrum β-lactamase–producing *Klebsiella pneumoniae* (n = 5, 41.7%) with intermediate sensitivity or resistance to imipenem and/or meropenem, *Acinetobacter* spp (n = 3, 25.0%; 2 *Acinetobacter baumannii* strains) resistant to imipenem and/or meropenem, *Pseudomonas aeruginosa* (n = 2, 16.7%) resistant to imipenem and meropenem, and extended-spectrum β-lactamase–producing *Proteus mirabilis* (n = 2, 16.7%) resistant to meropenem. Most XDR isolates were sensitive only to colistin or polymyxin B, neither of which is available in Iraq. Therefore, the only treatment option was multiple rounds of surgical debridement and wound care. The infection was deemed cured before discharge in 7 patients (58.3%). Meanwhile, 4 patients (33.3%) were discharged with unhealed wounds, and outpatient follow-up was planned. One patient died in the intensive care unit of a referral hospital after developing septicemia postsurgery. XDR bacteria pose substantial health risks in Iraq. Thus, improving antimicrobial stewardship and accessibility to essential antibiotics is critical to address this issue.

Antimicrobial resistance (AMR), an increasing global health threat [1, 2], is prevalent in patients with surgical trauma wounds in conflict-affected settings such as Mosul, Iraq [3–6]. Patients with surgical trauma, particularly those in low-resource and conflict-affected areas, face severe consequences when wounds become infected by drug-resistant bacteria, as infection leads to increased mortality, prolonged hospital stay, and severe economic losses for the patient and health systems [7–9]. A high prevalence of multidrug-resistant (MDR) infections has been identified in patients with trauma-related wounds [10]. In particular, extensively drug-resistant (XDR) bacteria represent a major concern. XDR infections have fewer treatment options, and such treatments are not easily accessible in low-resource settings [11].

Evidence on XDR infections in Iraq is scarce, with no reports from Mosul regarding the population with conflict-associated wounds [12, 13]. Therefore, this case series described the characteristics, clinical management, and outcomes of patients admitted with conflict-associated wounds complicated by XDR infections to the Médecins Sans Frontières (MSF) Tertiary Orthopaedic Care (TOC) center in East Mosul, Iraq, between May 2018 and February 2020.

## MATERIALS AND METHODS

### Setting

Mosul, which has been heavily affected by decades of conflict (most recently in 2017), has a deteriorated public health system [14]. MSF has been performing free orthopedic and reconstructive surgery since May 2018 at the TOC in East Mosul, adopting a multidisciplinary approach including surgery, physiotherapy, mental health care, and wound care. Infection prevention and control (IPC) measures, access to microbiology diagnostics, and antibiotic stewardship programs were established in response to the significant burden of MDR.

### Study Design and Population

This retrospective case series investigated the clinical and microbiological characteristics of patients admitted to MSF TOC in Mosul who were diagnosed with XDR infection between May 2018 and February 2020.

### Definition of XDR

XDR was defined as nonsusceptibility to at least 1 agent in all but ≤2 antimicrobial categories per the corresponding antibiogram, as stipulated by the international expert proposal for standard definitions for acquired resistance [15]. In microorganisms with intrinsic resistance to an antibiotic or antibiotic class, the antibiotic or class was removed prior to applying the XDR definition criteria. Only antibiotic families available within the country were tested. Therefore, the susceptibility of organisms to antibiotics that are not available in Iraq was not considered in the assessment of XDR resistance. This approach ensured that the classification of XDR was applicable and relevant to the treatments available in Iraq. If an antibiotic class was not tested, no assumption was made regarding bacterial susceptibility.

### Microbiological and Clinical Procedures

Antibiotic sensitivity testing was performed with the Kirby-Bauer disk diffusion method [16]. No other tests were performed. The interpretation was based on the guidelines of the Clinical and Laboratory Standards Institute [17] and performed in an external microbiology laboratory in Erbil (>80 km from Mosul). Extended-spectrum β-lactamase (ESBL) production was determined by detecting the synergy between clavulanic acid and a third-generation cephalosporin indicator. Testing was performed for all antibiotic classes available in Iraq. The guidelines do not have a breakpoint zone diameter for interpreting polymyxin B and colistin. In Iraq, testing for colistin was not available.

On admission, patients with signs of infection underwent biopsy at least 1 week after antibiotic washout following the MSF guidelines with clinical, blood, and radiologic examinations. In addition to the clinical assessment of the wound, the severity of the infection was assessed weekly by measuring C-reactive protein levels and the erythrocyte sedimentation rate. The technical procedures for debridement, wound care, and final suture after infection resolution followed the MSF guidelines [18].

### Data Collection

Information on XDR isolates was retrospectively collected from the WHONET microbiology laboratory database, which is routinely used in the TOC. Sociodemographic and clinical data were extracted from our routine database and patients’ medical records.

Patient outcomes were categorized as cured (no clinical infection signs, complete wound healing with no pus discharge, and normal C-reactive protein and erythrocyte sedimentation rate levels), uncured (outpatient follow-up required), or deceased at discharge.

## RESULTS

Between May 2018 and February 2020, 266 patients with a microbiologically confirmed infection were admitted to the MSF TOC in Mosul. More than half of these infections were caused by gram-positive bacteria (135/266, 50.8%), whereas the remainder were caused by gram-negative bacteria (131/266, 49.2%). Among the gram-negative bacteria, 67.9% (89/131) were ESBL producers. In total, 126 of 131 gram-negative bacteria were tested for carbapenem resistance with imipenem or meropenem, revealing that 23.8% of the isolates (30/126) were carbapenem resistant. Of the total patient cohort, 4.9% (13/266) were infected by an XDR organism, comprising 9.9% (13/131) of all gram-negative infections, and all of these organisms were gram negative. Twelve patients were included in this case series, and their characteristics are presented in Table 1. One patient was excluded because of incomplete records.

**Table 1.** Demographics and Clinical Characteristics of Patients With Extensively Drug-Resistant Infections Admitted May 2018–February 2020 in Mosul, Iraq

No.	Sex	Age, y	OPD Admission, No.	Nature of Injury	Site of Injury	Infection Type	Antibiotics Provided (All IV)	Treatment Duration, d	Comorbidities	Radiology	LOS, d	Debridement, No.	Outcome
1	M	24	10	Road traffic injury	Lower limb	Polymicrobial	Vancomycin, 1 g; imipenem, 1 g; gentamycin, 280 mg; colistin, 9 000 000-IU loading dose switched to 4 500 000	8	None	Osteomyelitis	30	14	Cured
2	M	30	NA	Bedsore	Back	Polymicrobial	Levofloxacin, 500 mg; amikacin, 750 mg	13	None	No bone involvement	45	3	Uncured
3	M	32	NA	Bedsore	Back	Polymicrobial	Amikacin, 1 g; ceftriaxone, 2 g	78	Neurologic disorder^a^	No bone involvement	120	3	Died
4	F	69	NA	Diabetic foot	Foot	Polymicrobial	Gentamycin, 160 mg; cefazolin, 1 g; metronidazole, 500 mg; ceftriaxone, 2 g	…^b^	Diabetes	Osteomyelitis	44	8	Cured
5	M	14	NA	Bedsore	Lumbar area	Monomicrobial	None	…	None	No bone involvement	72	4	Uncured
6	F	34	1	Suicidal attempt	Forearm	Polymicrobial	None	…	None	No fracture or osteomyelitis	18	4	Cured
7	F	52	2	Blast injury	Iliac region	Polymicrobial	None	…	Diabetes and hypertension	Osteomyelitis	22	2	Cured
8	M	17	NA	Shell injury	Sacrococcygeal	Polymicrobial	Imipenem, 500 mg; vancomycin, 1 g; amikacin, 1 g	8	Neurologic disorder^a^	No bone involvement	20	2	Uncured
9	M	29	8	Electrical burn	Hand	Polymicrobial	None	…	None	Foot amputation; no bone involvement	45	5	Cured
10	M	8	20	Road traffic injury	Foot	Monomicrobial	None	…	None	No fracture or bone involvement	22	7	Cured
11	M	42	19	Diabetic foot	Thigh	Monomicrobial	None	…	Diabetes and hypertension	No bone involvement	189	9	Cured
12	M	13	7	Bedsore	Lumber back	Polymicrobial	None	…	Neurologic disorder^a^	Osteomyelitis	9	3	Uncured

Abbreviations: d, days; F, female; IV, intravenous; LOS, length of stay; M, male; NA, not applicable; OPD, outpatient department; y, years.

^a^Paraplegia or spinal cord injury.

^b^Duration of antibiotic treatment missing for patient 4.

The median age of patients in the case series was 29.5 years (IQR, 15.5–38). They were predominantly male (n = 9, 75.0%). Six patients (50.0%) reported comorbidities. The median hospital stay was 37 days (IQR, 21–58.5), and patients underwent a median of 4 operations (IQR, 3–7.5) during their stay. All patients presented with clinical signs of infection on admission. Most injuries (n = 9, 75.0%) resulted from nonviolent trauma, whereas 3 (25.0%) resulted from violent trauma, namely blast injuries. The limbs were the most prevalent site of injury (n = 7, 58.3%). Most patients had soft tissue infections (n = 7, 58.3%), and 5 (41.7%) also had bone involvement (Table 1).

Of the episodes, 9 (75.0%) were polymicrobial and 3 (25.0%) were monomicrobial. At least 1 XDR isolate was identified in each episode. In addition to other transmission-based precautions, all patients were placed in single-bed isolation rooms.

**Figure 1.**
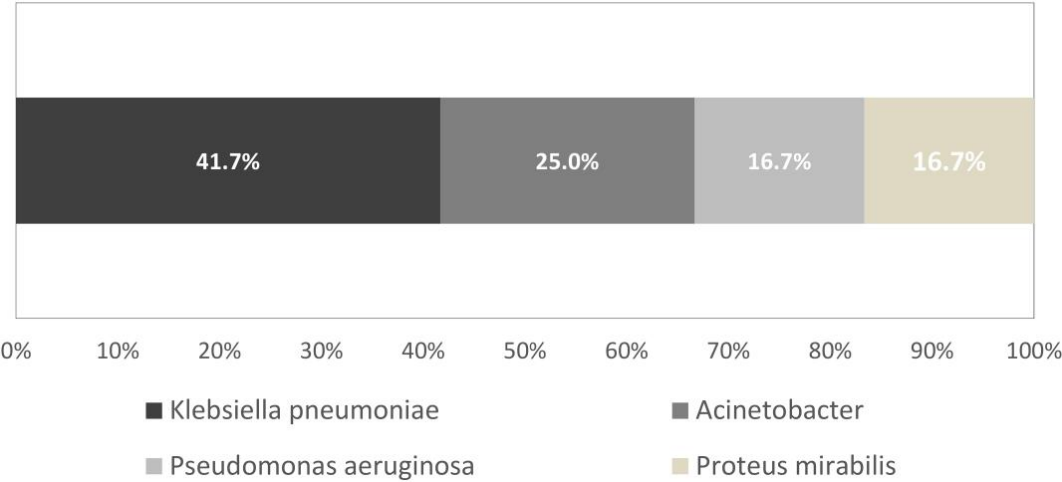
Type and proportion of bacteria isolated in bone biopsies from patients with extensively drug-resistant infections in Mosul, Iraq, May 2018−February 2020.

The most prevalent XDR organism was ESBL-producing *Klebsiella pneumoniae* (n = 5, 41.7%), exhibiting resistance or intermediate sensitivity to imipenem and/or meropenem. Other XDR isolates included *Acinetobacter* spp (n = 3, 25.0%), with resistance to imipenem and/or meropenem; *Pseudomonas aeruginosa* (n = 2, 16.7%), showing resistance to imipenem and meropenem; and ESBL-producing *Proteus mirabilis* (n = 2, 16.7%), with meropenem resistance (Figure 1). All isolated XDR organisms were gram negative. In polymicrobial episodes, additional pathogens included methicillin-resistant *Staphylococcus aureus*, *Enterococcus* spp, *Escherichia coli*, viridans streptococci, and *Burkholderia cepacia*.

Antibiotic susceptibility testing identified colistin and polymyxin B as the only potential treatments for XDR infections; however, neither drug is available in the country. Tigecycline resistance was noted in 71.4% (5/7) of tested isolates. Newer antibiotics, such as ceftazidime-avibactam or meropenem-vaborbactam, are not on MSF's essential medications list because of economic barriers and a lack of local efficacy data, resulting in reliance on surgical management, primarily debridement and subsequent wound care.

Seven patients (58.3%) were treated solely by surgical debridement. All patients who received antibiotics had polymicrobial infections. Antibiotics included ceftriaxone, amikacin, levofloxacin, gentamycin, and imipenem, and they were provided as slow infusions coupled with renal and liver monitoring to prevent side effects. One case required amputation of the lower limb.

The wounds of 7 patients (58.3%) were healed before discharge, and 4 patients (33.3%) were discharged with unhealed wounds and ongoing outpatient follow-up. One patient transferred to intensive care died from postoperative septicemia.

## DISCUSSION

Our study highlights the challenges of XDR infections in Mosul, Iraq, where specific antibiotic treatment options remain scarce [19], contributing to the global burden of AMR [20].

Data paucity on XDR infections in conflict-affected areas such as the Middle East [10, 21]—worsened by conflict-driven AMR contributors, such as collapsed health systems, limited staff training, restricted availability and misuse of antibiotics, inadequate IPC measures, and poor diagnostic capacity—exacerbates AMR issues [21–23]. This series highlighted the imminent threat of XDR gram-negative bacteria, recognized as critical pathogens of global health priority according to the World Health Organization, in Mosul, Iraq [24].

Of particular concern is the impact of XDR infections on the young study population, potentially resulting in prolonged disability during their peak productive years, thereby emphasizing the urgent need for (1) strengthened AMR surveillance in Iraq to address the emergence of XDR infections and contain transmission, (2) context-adapted IPC measures, and (3) improved diagnostic capabilities for better antimicrobial stewardship.

In Mosul, colistin and polymyxin B were often the only treatment options for some XDR infections. Regarding non–*Pseudomonas aeruginosa* infections, tigecycline, which is often not available for testing in external laboratories, was an option; however, it was not accessible because of supply shortages. The scarcity of these drugs alongside tigecycline resistance severely restricted therapeutic options. Therefore, access to essential antibiotics such as colistin and tigecycline [11, 25], which are recognized as last-resort antibiotics against MDR pathogens [26], is critical for the treatment of XDR infections caused by gram-negative bacteria in Iraq [27]. Novel antibacterial agents such as β-lactam/β-lactamase inhibitor combinations, representing promising alternatives against MDR and XDR infections [28], have not yet been added to MSF's essential medication list because the local resistance patterns, which are critical for selecting relevant antibiotics, have not been clarified. The high costs associated with these new antibiotics, which are up to 20-fold more expensive than older alternatives for carbapenem-resistant Enterobacterales, MDR *Pseudomona aeruginosa*, and carbepenem-resistant *Acinetobacter baumannii* [29], represent a significant barrier to accessibility. Despite potential availability through MSF support, ensuring a continuous and sustainable supply of these expensive drugs in low-resource settings remains challenging. Considering the growing issue of AMR worldwide, there is a pressing need to ensure access to established and emerging antibiotics in low-resource settings to effectively manage infections and save lives.

Although we advocate for improved antibiotic access, we emphasize the need for alternative strategies such as surgical debridement and subsequent wound care for managing AMR infections in extreme situations in which necessary antibiotics are unavailable. Although this approach provides effective wound healing, as presented in this study, aiming at definitive source control might be insufficient to treat complex infections such as osteomyelitis [30], in which concurrent targeted antibiotic therapy is often necessary to prevent early relapse and complications. This study did not monitor whether cured patients who were released without further antibiotic therapy later experienced relapse.

Beyond antibiotic treatment and surgery, quality IPC measures such as placing patients in single-room isolation are essential in this context to prevent XDR transmission within the facilities. The identification of XDR cases in an MSF facility highlights the broader public health concern of XDR pathogen circulation in the community and other facilities in Mosul, particularly when surgical care is the only option amid limited antibiotic accessibility.

## CONCLUSION

XDR infection poses a substantial challenge in Mosul, Iraq. XDR infection management in this setting highlights the critical role of surgical management in a context in which access to essential antibiotics is problematic. Although larger studies are needed to clarify the actual prevalence of XDR infection in Mosul, improved accessibility to older and newer antibiotics is necessary to ensure appropriate XDR infection management and prevent further transmission.

## Notes

***Acknowledgments.*** We thank the MSF TOC staff for their continuous work toward improving patients’ outcomes, as well as our patients who trusted us throughout their clinical management. This series report was produced as part of the activities of the TOC center of MSF in Mosul, Iraq, which is maintained with MSF funding.

***Author contributions*.** E. A. and K. M. conceptualized the idea. All authors contributed to the write-up of the study protocol, including design and methodology. H. A. A., H. H. M., H. H. A. S., and A. N. T. supported the collection of the data. H. A. A. and K. M. were responsible of data curation. Analysis of the results was done by H. A. A. with the support and validation of A. W. and K. M. All authors discussed and supported the interpretation of the results. H. A. A. drafted the original manuscript with the support of P. G.-V. and K. M. K. M. supervised the project. All authors reviewed the results and approved the last version of the manuscript.

***Data availability*.** Data presented in this study are not available publicly but upon request to interested researchers due to confidentiality and contextual privacy concerns.

***Patient consent statement*.** This research was approved by the MSF Ethics Review Board under protocol 2086 (16/12/2020) and by the Ministry of Health and Environment of the Nineveh Health Directorate in Iraq under protocol number 31210 (08/12/2020). All patient data used in this study were pseudoanonymized and did not include direct patient identifiers. Written consent from patients was waived by the MSF and local ethics committees following the nature of the study design.

***Financial support*.** This study did not receive any funding. The work was supported by Médecins Sans Frontières.
